# An interview with Benedict Wilmes

**DOI:** 10.1590/2177-6709.21.6.026-033.int

**Published:** 2016

**Authors:** Guilherme Thiesen, Marcus Vinícius Neiva Nunes do Rego, Jorge Faber, Ki Beom Kim

**Affiliations:** 1 » Post-doctoral Fellow in Orthodontics, Center for Advanced Dental Education, Saint Louis University, Saint Louis, MO, USA. » DDS, MSc and PhD in Orthodontics and Dentofacial Orthopedics (UFSC, PUCRS and ULBRA). » Diplomate, Brazilian Board of Orthodontics and Dentofacial Orthopedics. » Professor of Orthodontics, UNISUL, Santa Catarina, Brazil.; 2 » DDS, MSc and PhD in Orthodontics and Dentofacial Orthopedics (UFPI, PUCRS and SLMandic). » Professor of Orthodontics, UNINOVAFAPI, Piauí, Brazil. » Professor of the Postgraduate Orthodontic Program, UFPI, Piauí, Brazil. » President of the Brazilian Association of Orthodontics, section Piauí (ABOR/PI).; 3 » Editor-in-chief, Journal of the World Federation of Orthodontists. » Adjunct Professor of Orthodontics, University of Brasilia, Brazil. » Former editor-in-chief, Dental Press Journal of Orthodontics. » Board Certified, Brazilian Board of Orthodontics and Dentofacial Orthopedics. » WFO fellow. » Member of the Associação Brasileira de Ortodontia (ABOR).; 4 » Associate Professor, Department of Orthodontics, Center for Advanced Dental Education, Saint Louis University, Saint Louis, MO, USA. » DDS, MSD, PhD (Dankook University, South Korea and Vanderbilt University, Nashville, TN, USA). » Diplomate, American Board of Orthodontics. » Diplomate, American Board of Orofacial Pain.

**Figure:**
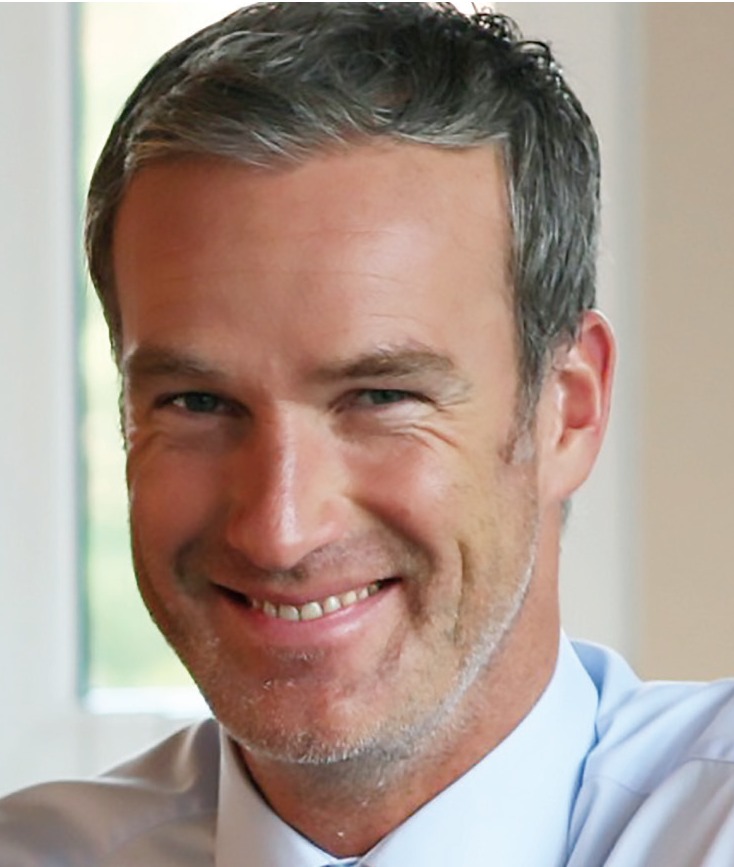

It is a great pleasure to bring to the readers of Dental Press Journal of Orthodontics some of the clinical and scientific knowledge from this great German orthodontist: Prof. Dr. Benedict Wilmes. Dr. Wilmes was raised in Soest, a small village with 50,000 inhabitants in the middle of Germany. He attended Dental School in Muenster, a nice university city near Netherlands. He first received a post-graduate degree in Oral Surgery at the Department of Maxillofacial Surgery at University of Muenster, and subsequently he did a post-graduation in Orthodontics and Dentofacial Orthopedics at the University of Duesseldorf. Dr. Wilmes has published more than 100 articles and textbook chapters. His primary interest is in the area of non-compliant and invisible orthodontic treatment strategies (TADs, lingual Orthodontics and aligners). His favorite hobbies are sports and philosophy. He even was a professional basketball player for the 1st and 2nd divisions in Germany. Lastly, I would like to disclose my gratitude to the DPJO for the opportunity of this interview, to the professors who contributed with the questions, and especially to Dr. Wilmes, who shared his experience and let us know a little more about his brilliant work. *Vielen Dank*!

Guilherme Thiesen - interview coordinator 

É uma satisfação imensa trazer aos leitores do *Dental Press Journal of Orthodontics* um pouco do conhecimento clínico e científico deste grande ortodontista alemão: Prof. Dr. Benedict Wilmes. Dr. Wilmes foi criado em Soest, uma pequena vila com 50.000 habitantes no meio da Alemanha. Ele é formado em Odontologia pela *University of Muenster*, uma acolhedora cidade universitária alemã perto da divisa com a Holanda. Sua primeira pós-graduação foi em Cirurgia Oral no Departamento de Cirurgia Maxilofacial da *University of Muenster* e, posteriormente, ingressou na pós-graduação de Ortodontia e Ortopedia Facial na *University of Duesseldorf*. Dr. Wilmes já publicou mais de 100 artigos e capítulos de livros. Sua principal área de interesse é em estratégias que não dependam da colaboração dos pacientes, bem como no tratamento ortodôntico com aparelhos invisíveis (DATs, Ortodontia Lingual e alinhadores). Seus passatempos favoritos são os esportes e a filosofia. Ele foi, inclusive, jogador profissional de basquete, pela 1ª e 2ª divisões do campeonato alemão. Por fim, eu gostaria de expressar minha gratidão ao DPJO pela oportunidade dessa entrevista, aos professores que contribuíram com suas perguntas e, especialmente, ao Dr. Wilmes, que compartilhou sua experiência e nos permitiu conhecer um pouco mais do seu brilhante trabalho. *Vielen Dank!*

Guilherme Thiesen - coordenador da entrevista 

## What are the advantages of the mechanics for upper-molar intrusion you have developed (Mousetrap Mechanics) compared with other conventional mechanics for molar intrusion? Is there a limitation for intrusion? If simultaneous intrusion of the first and second upper molars is needed, what variations in appliance design and/or force system do you use? Marcus Vinicius Neiva Nunes do Rego

The mostly used insertion site of miniscrews is in the alveolar process. However, there are a number of disadvantages related to the insertion into the interradicular area of the upper molars:

»There is often insufficient space on the buccal aspect to insert a miniscrew safely between tooth roots.^1^ ^-^ ^3^
»The periodontal structures may be damaged if the miniscrew contacts the surface of a tooth root and the risk of failure of the miniscrew will be higher.^4^ ^,^ ^5^
»The reduced interradicular area on the buccal alveolar process of the upper molars limits the placement of miniscrews to those with a small diameter.^6^ However, small diameters are associated with a higher risk of fracture^7^ and failure.^8^ ^-^ ^10^
»Intrusive movement may be stopped and the root surface may be damaged when a molar is moved directly against a mini-implant during intrusion.^11^ ^,^ ^12^
»There is risk of penetration of the maxillary sinus when a miniscrew is inserted into the posterior area of the upper alveolar process.^13^

To minimise insertion risks, a prudent strategy is the placement of miniscrews safely away from the roots and the teeth to be moved. The anterior palate provides for a suitable alternative insertion site where miniscrews with larger dimensions and higher stability^14^ ^,^ ^15^ may be placed in a region with a high bone quality, thin overlying soft tissue and negligible risk of causing interference with nearby teeth.^16^

**Figure 1:**
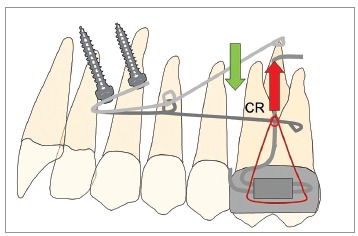
"Mousetrap" mechanics for upper molar intrusion using TADs in the anterior palate. If the molars should be just intruded, the line of force must pass through the estimated center of resistance (CR).

**Figure 2:**
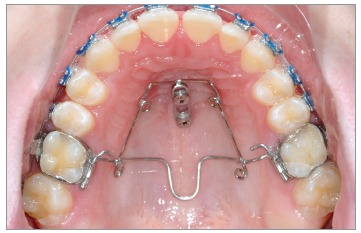
"Mousetrap" mechanics for upper molar intrusion using TADs in the anterior palate in an open bite case.

To conclude: every strategy has pros and cons. Advantages of the Mousetrap are a safe insertion site for the Temporary Anchorage Devices (TADs) and a constant and predictable level and direction of forces (Figs 1 and 2). Disadvantage might be the bigger dimension of the appliance. 

I don't know if there is a limit of intrusion, we have intruded some molars around 4-5 mm. However, the risk of root resorption and the soft tissue excess after a distinctive intrusion have to be considered. 

If more than one tooth in a quadrant is to be intruded, teeth can be coupled before intrusion. As an alternative, a two stage intrusion can be performed: 1) Intrusion of one molar; 2) Levelling and intrusion of adjacent teeth. Both strategies are possible.

## Since your TADs anchored distalizer for Class II correction (named Beneslider) applies forces on the palatal surfaces of the molars, and a common characteristic of Class II malocclusions is a mesial rotation of the molars, how do you usually control this aspect? Guilherme Thiesen

**Figure 3:**
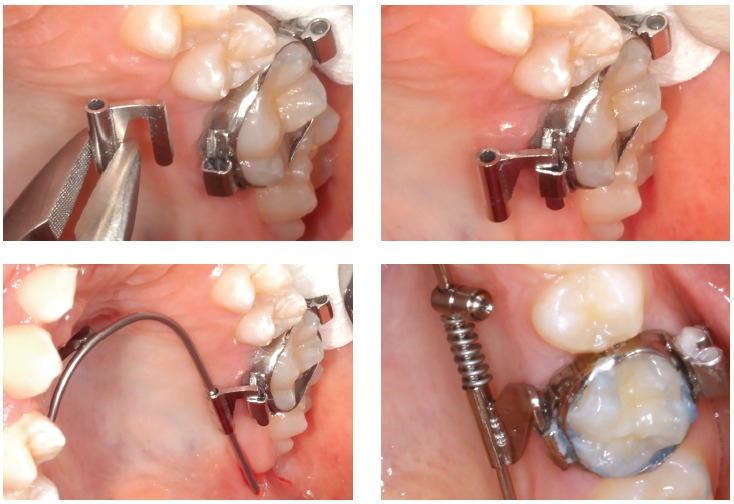
Chairside adaptation of a Beneslider appliance. Due to prefabricated parts, impression and laboratory procedure are not needed.

**Figure 4:**
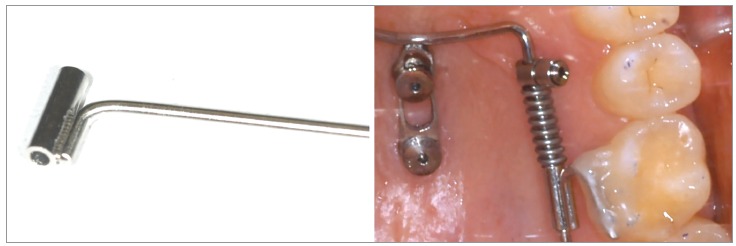
If bands are not used, a Benetube according to Dr. Banach may be used.

From my point of view, there are three key points to avoid molar tipping and rotation during distalization: 1) a safe source of anchorage; 2) a rigid guiding wire (1.1 mm in the Beneslider) and 3) a rigid coupling with the molars to be distalized. However, we see sometimes a little bit of rotation due to the little play using the molar sheath and the conventional Benetube (Fig 3). A more rigid coupling from the Beneslider to the molars is obtained in the bonded Benetube (acc. to Dr. Banach, Fig 4). 

## You usually demonstrate in your lectures some different designs of molar mesialization appliances (T-wire, Mesialsliders, etc.). What are the clinical differences between them? I mean, when do you indicate one or another? Ki Beom Kim

**Figure 5:**
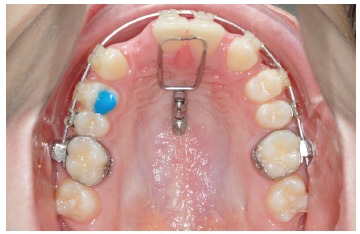
T-wire for indirect anchorage of the anterior dentition. Space closure to the mesial was conducted.

**Figure 6:**
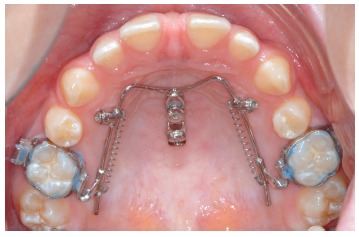
Mesialslider for mesialization of the upper molars (direct anchorage).

If the central incisors are in the correct position (midline, torque and angulation is correct), a T-wire^17^ (Fig 5) can be bonded to the lingual surfaces of the central incisors to apply an indirect anchorage with the goal of avoiding lingual tipping of the central incisors during space closure.^17^ ^-^ ^19^ As an alternative to the T-wire (indirect anchorage), the Mesialslider^17^ ^,^ ^18^ (Fig 6) as a direct anchorage device can be used. The use of the T-wire leads to a very easy mechanics, but the Mesialslider has some advantages: 1) Since the incisors are not fixed, a midline deviation and incorrect dental torque can be adjusted at the same time. 2) Brackets are not needed during the use of the Mesialslider (and Beneslider), what makes this phase of the treatment much more comfortable for the patient. 

## On the AAO meeting held in Orlando in 2016, you demonstrated a lot of cases in which you combined the Beneslider system with Invisalign treatment after that. Can you describe it better how to manage that? How can we use these appliances for anchorage after achieving the desired distalization of the molars? Guilherme Thiesen

**Figure 7:**
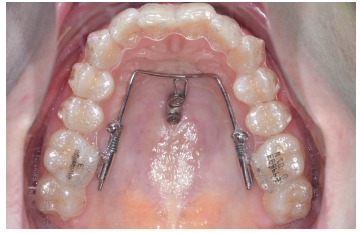
Combination of the Beneslider and aligner: After distalization with the Beneslider, aligners are used for finishing of the case. The Beneslider might stay in place passively for molar anchorage.

In the US and in Europe, the use of aligners became very popular over the last decade. At the University of Düsseldorf, we are following this two-step strategy: 1) Moving the upper molars (and premolars) with (Bene-) or Mesialsliders and 2) Taking an impression and finishing the case with aligners.^20^ I think this is a great option for esthetic and non-compliant Orthodontics. In phase 2, we leave the Beneslider in place for anchorage purposes (Fig 7). 

## Nowadays, what are the biggest challenges you face when treating a malocclusion with aligners? Which are your criteria for Invisalign indication? Do you overcorrect some movements? Guilherme Thiesen

I think that bodily sagittal movements and vertical movements are very difficult with aligners. Thus, we can broaden the treatment opportunities by adding TAD borne sliders for bodily movements or the "Mousetrap" for molar intrusion in the upper arch. If there is a difficult treatment task in the lower arch, I am still choosing fixed appliances.

## Most of the miniscrews for your mechanics are placed in the anterior palatal region. Some of them are inserted right at the midpalatal suture. Do you have any concerns about placing miniscrews into the suture especially in adolescent patients? Ki Beom Kim

The clinician has to differentiate between a median and paramedian pattern of location of miniscrews. There is no difference in regards to the continued retention and stability of miniscrews between median and paramedian insertion, even among children and adolescents.^21^ ^,^ ^22^ The possibility of growth impairment due to the location of implants within the midpalatal suture was investigated by Asscherickx et al,^23^ who inserted two dental implants (Straumann palatal implant) in the suture of Beagle dogs and discussed a transversal growth inhibition of the maxilla. However, in this study, only one control animal was available and only one parameter was found to be different.^24^ Secondly, the transferability of findings from this study to miniscrews is questionable, due to the greater diameter and the surface roughness of the dental implants. Clinical observations at our Institution have not revealed a tendency of impaired transversal growth of the maxilla. As such, the clinically relevant impairment of maxillary growth due to a median inserted miniscrew seems unlikely. Contrastingly, a median insertion is considered to be advantageous due to the profound reduction in risk of injury to the roots of the upper incisor teeth, during the insertion procedure. 

## Therefore, what are the most important details related to TADs insertion on the anterior palate? Jorge Faber

**Figure 8:**
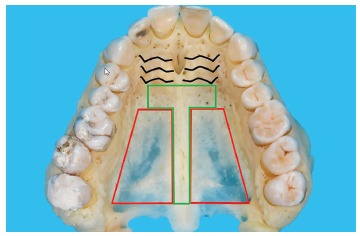
The T-Zone (green) is indicating the recommended insertion site for palatal TADs. In the posterior-lateral area (red) the available bone is very thin.

Very easy: Stay in the T-Zone posterior from the rugae (green area, Fig 8). Avoid the posterior lateral area, due to lack of bone (red area, Fig 8). 

## A study you published in 2015 **^25^** , in which you compared the classic maxillary protraction protocol with another protocol using the Hybrid Hyrax appliance (anchored on TADs placed in the palate), showed less forward movement of the maxilla and improvement of maxillomandibular relationship, if we compare your results with the findings reported by Hugo De Clerk's miniplate approach. What is the reason for this difference? Marcus Vinicius Neiva Nunes do Rego

Mostly, it doesn't make sense to compare these values from different studies. Maybe there are many reasons for bias due to the different choice of patients in the different institutions etc. We need RCTs in the future to be able to compare these treatment approaches. 

**Figure 9:**
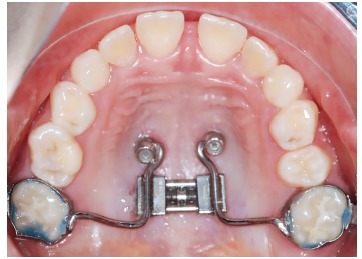
Hybrid Hyrax with two TADs in the anterior palate for rapid maxillary expansion and early Class III treatment.

**Figure 10:**
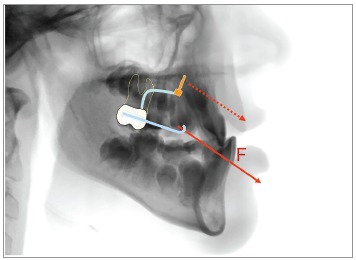
Principle of the Hybrid Hyrax facemask combination: The force is transferred to bony structures, minimizing dental side effects.

Clinically, we tried to be as less invasive as possible. That was the reason to use the Hybrid Hyrax with just two miniscrews instead of two miniplates for pure skeletal protraction of the maxilla without dental side effects (extrusion and mesial migration of the molars, Figs 9 and 10). 

## The Mentoplate is an innovative method for Class III treatment in growing patients. What are the main advantages of this technique in comparison to Hugo De Clerk's miniplate approach? Jorge Faber

**Figure 11:**
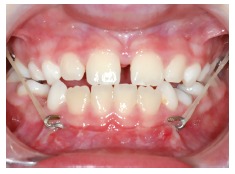
TAD borne early Class III treatment: Intraoral elastics are attached to the Mentoplate and to the bands of the Hybrid Hyrax.

**Figure 12:**
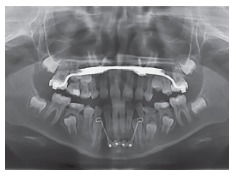
The Mentoplate is inserted in the mental area, with an outstanding bone quality. Insertion is possible at the best age for orthopedic treatment (before puberty, 8-10 years of age).

**Figure 13:**
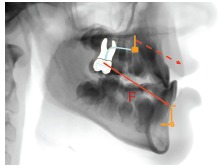
Principle of the Hybrid Hyrax-Mentoplate combination: The force is transferred to bony structures minimizing dental side effects. Hence, an extraoral device could be avoided.

First of all, I really admire Hugo De Clerk's work. From my point of view, he had many outstanding ideas and he is for sure one of my role-models. However, I think there are several advantages of the Mentoplate and Hybrid Hyrax (Figs 11-13) compared to the Bollard miniplates. 

First of all, the Bollard miniplates cannot be inserted before the lower canines are erupted (around 12 or 13 years old). As a consequence, the patient is, according to many studies (eg. from Lorenzo Franchi^26^ ^)^, beyond the best age for an orthopedic Class III treatment. The Mentoplate can be inserted very early, our favorite age is around 8-9 years old. 

Secondly, we are missing the "RPE-effect" and the "Alt-RAMEC-effect" with stimulation of midface sutures for bigger maxillary protraction. We know that this stimulation results in more protraction of the maxilla.^26^

Thirdly, the palatal TADs are less invasive and more stable than upper miniplates since the failure rate of the palatal TADs is almost zero.^27^

## In the treatment of Class III malocclusion with skeletal anchorage, do you believe that a rapid maxillary expansion prior to traction is needed even when there is no transverse discrepancy? Marcus Vinicius Neiva Nunes do Rego

RME is not needed, but it improves the skeletal effects of the Class III therapy, especially using Alt-RAMEC (see previous question). 

## In some parts of the world, such as in Brazil, parents tend to refuse procedures under general anesthesia. At the same time, in growing Class III patients, miniplates are very often placed under general anesthesia. How well do European parents accept this anesthetic protocol, and what is your point of view about the surgical risks and benefits of miniplate treatment in growing Class III patients? Jorge Faber

I think, there is not a big difference between parents around the world. All parents want to do the best for their children. Of course, we have to talk about risks and benefits for all our treatments and let the parents and patients make the final decision. The risks of miniplates are very low if they are placed away from roots and nerves. This may be another advantage of the Mentoplate, it is inserted in a very safe area, away from the roots. 

## There are many case reports using the miniscrews in the buccal shelf or retromolar region to distalize the entire mandibular dentition to correct Class III malocclusions. What is your opinion about this type of mechanics? Do you have any suggestions for Class III malocclusion with true prognathic mandible besides using miniscrews or miniplates in the mandibular anterior area and infrazygomatic area? Ki Beom Kim

I don't think that there are so many indications for lower distalization, especially in Europe and the US. There is always the risk that there is no space distally, and therefore the distalization of the lower dentition will be a difficult task.
